# Medical emergencies in pediatric blood & marrow transplant and cellular therapies

**DOI:** 10.3389/fped.2023.1075644

**Published:** 2023-02-07

**Authors:** Nikki Agarwal, Seth Rotz, Rabi Hanna

**Affiliations:** Department of Pediatric Hematology, Oncology and Bone Marrow and Blood Transplant, Cleveland Clinic, Cleveland, OH, United States

**Keywords:** pediatric bone marrow transplant, emergencies, cellular therapy, transplant-related morbidity, life-threatening

## Abstract

Hematopoietic stem cell transplant (HCT) is used for many pediatric malignant and non-malignant diseases. However, these patients are at a high risk for emergencies post-transplant, related to prior comorbidities and treatments for the underlying disease, high dose chemotherapy regimen related toxicities, prolonged myelosuppression, and opportunistic infections due to their immunocompromised state. Emergencies can be during preparative regimen and hematopoietic progenitor cell (HPC) infusion, acute post-transplant (pre-engraftment) and late during post engraftment. Infectious complications are the most common cause of morbidity and mortality in the peri-transplant period. Sinusoidal obstructive syndrome is another life-threatening emergency seen in children undergoing HCT, especially in infants. Timely recognition and administration of defibrotide with/without steroids is key to the management of this complication. Another complication seen is transplant associated thrombotic microangiopathy. It can cause multiorgan failure if left untreated and demands urgent identification and management with complement blockade agents such as eculizumab. Cytokine release syndrome and cytokine storm is an important life-threatening complication seen after cellular therapy, and needs emergent intervention with ICU supportive care and tocilizumab. Other complications in acute period include but are not limited to: seizures from busulfan or other chemotherapy agents, PRES (posterior reversible encephalopathy syndrome), diffuse alveolar hemorrhage, idiopathic pulmonary syndrome and allergic reaction to infusion of stem cells. Acute graft versus host disease (GvHD) is a major toxicity of allogeneic HCT, especially with reduced intensity conditioning, that can affect the skin, liver, upper and lower gastrointestinal tract. There has been major development in new biomarkers for early identification and grading of GvHD, which enables application of treatment modalities such as post-transplant cyclophosphamide and JAK/STAT inhibitors to prevent and treat GvHD. Myelosuppression secondary to the chemotherapy increases risk for engraftment syndrome as well as coagulopathies, thus increasing the risk for clotting and bleeding in the pediatric population. The purpose of this article is to review recent literature in these complications seen with pediatric hematopoietic cell transplant (HCT) and cellular therapies and provide a comprehensive summary of the major emergencies seen with HCT

## Introduction

Over the past 60 years, there have been remarkable advances in hematopoietic stem cell transplant (HCT) and cellular therapies. Use of HCT has increased from the first transplant in 1957, to ∼10,000 by 1985, and more than 1 million in 2012 (1). Multiple studies have identified different sources for stem cells, and conditioning treatments have been tailored to decrease graft rejection and transplant related mortality. Newer immune therapies are being introduced to facilitate tolerance between donor and recipient as well as to decrease graft versus host disease (GVHD) and improve overall survival (2, 3). HCT can be a lifesaving treatment, but it comes with considerable risks that can be life-threatening. Aside from the risk of relapse, HCT can be associated with significant early and late treatment related mortality (TRM). Infections, toxicity from high dose chemotherapy/radiation and GVHD (in allogeneic transplants only) are the main causes of death (4). The overall risk of non-relapse mortality after allogeneic HCT has decreased from 27% in the 1990s to 11% in 2010–2016 (3), likely from better patient and donor selection, improved supportive care for infections as well as use of the hematopoietic cell transplant-comorbidity index (HCT-CI) to predict TRM and choosing appropriate conditioning therapy regimen. This scoring system has been validated in various retrospective and prospective studies worldwide and has identified risk for non-relapse mortality (NRM) associated with HCT (5–7). It is determined by end-organ function, performance score, history of cardiac, pulmonary, or renal comorbidities and diabetes.

Medical emergencies may occur throughout HCT. During conditioning therapy, complications may arise from high dose chemotherapy and radiation. After the stem cell infusions, patients are immunocompromised for a period of time which increases risk for opportunistic infections and emergencies related to the pancytopenia. The innate immunity recovers within the first few weeks post-transplant, while the adaptive immunity may take up to two years or longer to recover fully (8). Even though patients may recover their neutrophil counts in the first few weeks post HCT, the neutrophils may be dysfunctional for up to 2 months post-transplant, and in presence of invasive fungal infections, neutrophil function recovery can take more than 6–12 months (Table 1).

**Table 1:** Adapted from Ogonek et al (65).

Type of immune cells	Recovery after allogeneic HCT
Neutrophils	∼14–21 days, can be up to 6 months
NK cells	Up to 100 days
T-cells	100 days – 2 years
CD19+ B cells	Up to 2 years

Other complications that can be seen in the immediate post-transplant phase arise from the systemic effect of chemotherapy and the immune interaction between donor and recipient cells on different organs. These can include neutropenic colitis, sinusoidal obstructive syndrome (SOS), posterior reversible encephalopathy syndrome (PRES), and transplant associated thrombotic microangiopathy (TA-TMA).

### Emergencies associated with HCT and cellular therapy

During HCT and cellular therapies, there are common complications (Table 2) which can be life threatening if not identified and managed in a timely manner. During the pre-transplant period, a preparative regimen with high-dose chemotherapy, with or without radiation, can cause severe myelosuppression and increase risk for opportunistic infections. Severe sepsis can be five-fold higher in HCT recipients compared to non-HCT recipients (9). Allergic/anaphylactic reactions are another acute complication seen with the preparative regimen, especially in association with anti-thymocyte globulin or alemtuzumab, common medications used to prevent GVHD and graft rejection. Cell infusions can also cause anaphylaxis and dimethyl sulfoxide (used to cryopreserve cells) may cause dysrhythmias. Prior to engraftment, HCT recipients are severely immunocompromised and at very high risk for serious bacterial, viral, and fungal infections. The high volume of fluids given with the high-dose chemotherapy and the cell infusions increases the risk for fluid overload and hypertension. This can predispose to seizures and posterior reversible encephalopathy, especially with the concomitant use of calcineurin inhibitors (CNI) such as tacrolimus to prevent GVHD. If these patients present with acute septic shock, it is important to limit fluid resuscitation and initiate early use of vasopressors to avoid the risk of fluid overload and pulmonary edema. High-dose cyclophosphamide (Cy) use in the preparative regimen can induce acute cardiotoxicity and can manifest with endothelial injury, arrhythmias, and fatal myopericarditis. In a large cohort study including 811 patients undergoing HCT and receiving cumulative Cy doses of >100 mg/kg, 12 patients (1.5%) developed cardiac failure (10). Endothelial disorders post-HCT include sinusoidal obstructive syndrome and thrombotic microangiopathy. The use of cellular therapy has been associated with cytokine release syndrome and immune effector cell-associated neurotoxicity syndrome (ICANS), which can be life-threatening if left unidentified. Post-engraftment, GVHD can be acute or chronic and involve multiple organs such as the skin, liver, gut, lungs, and other organs. In the following few sections, we will detail some of the more common complications and review recent advances in their management.

**Table 2:** Transplant related emergencies and morbidities.

1. Infections:	Sepsis (encapsulated bacteremia, staphylococcus, and streptococcus) HHV6 HSV Adenovirus Disseminated varicella infection CMV Pneumocystis jirovecii (PJP) Invasive fungal infections
2. Gastrointestinal:	Mucositis Typhilitis/neutropenic colitits Gastrointestinal bleeding Sinusoidal obstructive syndrome
3. Neurological:	Intracranial hemorrhage PRES Drug toxicity Seizures Immune effector-cell associated neurotoxicity syndrome (ICANS)
4. Immunological:	Acute GVHD (gastrointestinal, skin, lungs, liver, kidney) TA-TMA (CNS, kidney, lungs) Sinusoidal obstructive syndrome (liver) Cytokine release syndrome (CRS) Allergic reaction/anaphylaxis
5. Cardiac:	Cardiomyopathy Arrhythmias Myocardial necrosis Pericardial effusion
6. Pulmonary:	Diffuse alveolar hemorrhage Idiopathic pulmonary syndrome Bronchiolitis obliterans
7. Hematological:	Transfusion reactions Pancytopenia Bleeding Coagulopathies
8. Renal:	Fluid overload and hypertension Electrolyte disturbances GVHD

## Infections

Infections are the most common cause of morbidity and non-relapse mortality in allogeneic and autologous HCT. There is an increased risk for bacterial, viral, opportunistic, and fungal infections. Early recognition of sepsis/infections in HCT patients is critical. Management with supportive care, appropriate antibiotics, antivirals, or antifungals as needed is required to prevent morbidity and mortality. During the pre-engraftment period, due to neutropenia and barrier breakdown (mucositis and central lines), there is increased incidence of bacterial infections. Post-engraftment, viral and fungal infections are more common due to impaired cellular and humoral immunity especially if patient develops GVHD requiring further systemic immunosuppression. Other factors determining risk for infection include history of infection with invasive fungi, Epstein-Barr virus (EBV), cytomegalovirus (CMV), varicella zoster (VZV), herpes simplex virus (HSV) and adenovirus. An extensive infectious disease workup is performed prior to preparative chemotherapy, to help guide prophylactic and pre-emptive therapies (11–13).

### Bacterial infections

HCT recipients are exposed to bacterial infections due to severe immunosuppression as well as mucosal barrier breakdown. Pre-engraftment, they are at a high risk of gram-positive and gram-negative bacteremia, especially *Streptococcus viridans* and *Enterococcus fecium,* which is often vancomycin resistant (VRE). Gram negative bacteremia usually arises due to disruption of the GI mucosa by the preparative regimen, most commonly being *Enterobacteriaceae.* Use of prophylactic antibiotics such as fluoroquinolones have helped decrease the incidence of gram-negative bacteremia, however there is always concerns for changing the gut microbiome with use of prophylactic antibiotics, that can predispose these patients to drug-resistant bacterial infections. HCT recipients are at high risk for multiorgan dysfunction, including hepatic and renal dysfunctions, which increase their risk for post-engraftment bacteremia (14), most commonly with Coagulase negative *Staphylococcus* and VRE *Enterococcus.* Most gram-negative bacteremia post-engraftment is secondary to *Enterobacteriaceae*, *Pseudomonas, Acinetobacter,* and *Stenotrophomonas,* especially in presence of central vascular lines. Both early and late pneumococcal infections have been reported in HCT recipients, especially in the presence of active chronic GVHD (15). This is due to inadequate antibody production as well as functional hyposplenism seen in these patients.

### Pneumocystis jirovecii pneumonia (PJP)

It is a life-threatening complication, and hence patients undergoing HCT are placed on PJP prophylaxis with trimethoprim/sulfamethoxazole (TMP-SMX) or pentamidine. Cumulative incidence of PJP pneumonia post-transplant ranges between 0.6%–4%. A study from CIBMTR reviewed all recipients of HCT between 1995 and 2005 and noted the incidence of PJP in 0.63% allogeneic and 0.28% autologous HCT recipients. Cases occurred as early as 30 days to beyond a year after allogeneic HCT, and proportional hazards model revealed that patients with PJP infections were 6.87 times more likely to die versus matched controls (*p* < 0.0001). The study identified several risk factors for PJP disease, which have been previously reported as well, including corticosteroid exposure, *in vivo* or *in vitro* T cell depletion, lymphopenia, immunosuppression, and GVHD (16). Clinical manifestations can be non-specific such as progressive dyspnea, low-grade fever, cough, hypoxemia, and diffuse rales on exam. Diagnosis is primarily based on imaging (usually CT scan) and evaluation of respiratory samples for presence of PJP. Treatment requires appropriate supportive care, and use of anti-PJP medications, primarily TMP-SMX (17). Adjunctive steroids have been used in HIV patients with moderate or severe PJP and a meta-analysis (18) demonstrated the benefit of steroids to improve clinical outcomes and mortality in HCT patients with PJP infection, without increasing the risk of other opportunistic infections.

### HSV encephalitis

Herpesvirus infections can manifest as encephalitis/myelitis. It can occur in the early-stage post-transplant. Clinical manifestations include fever, malaise, headache, nausea followed by seizures, altered mentation and focal neurological deficits within 24 h of onset of symptoms. HSV encephalitis may present with aphasia and intracerebral hemorrhage in the post-transplant setting. There can be behavioral or personality changes. Fever may be absent due to immunocompromised state, and a high suspicion is needed for early diagnosis. Definitive diagnosis can be done by HSV PCR in blood and CSF. Management includes supportive care and intravenous high dose acyclovir in all suspected cases, as delaying institution of acyclovir for confirmatory tests increases risk for mortality.

### CMV disease

CMV reactivation is a major viral infectious complication after allogeneic HCT and is associated with an increased risk of non-relapse mortality (19). Advances in transplant and antiviral treatment, as well as widespread PCR monitoring and pre-emptive practices over the last 25 years have reduced the incidence of CMV disease to ∼10% in the first year post-transplant (20, 21). Uncontrolled viral replication is primarily seen in the first 100 days post-transplant. In a cohort of 86 PCR-monitored and preemptively treated patients, CMV disease in the first 100 days post-HCT was noted to be 3.5% and late onset CMV disease was seen in 6% (22). Risk is determined by pre-transplant serostatus of the recipient and the donor, grade of HLA disparity, use of T-cell depleted graft, presence of GVHD and ongoing use of immunosuppression. The rate of immune recovery influences the incidence of CMV. Uncontrolled CMV replication following reactivation can lead to life-threatening end-organ disease, commonly manifesting as interstitial pneumonia and gastrointestinal disease, and less frequently as retinitis, hepatitis and encephalitis (23). Treatment is with supportive care, antivirals, and newer modalities such as adoptive T-cell therapy.

### BK virus induced hemorrhagic cystitis

BK virus related hemorrhagic cystitis (HC) is a significant cause of morbidity post allogeneic HCT. The reported incidence is 8%–25% in the pediatric population (24). Risk factors include GVHD and use of cyclophosphamide in the preparative regimen as well as GVHD prophylaxis post-transplant. Primary infection with BK virus is common in childhood and is usually asymptomatic. However, during HCT, injury to bladder mucosa by the conditioning regimen as well as reactivation of latent BK virus secondary to immunosuppression cause BK viruria. After immune reconstitution post-engraftment, there is immune mediated urothelial injury due to the viruria, ultimately leading to HC. It usually presents between 2 weeks to 6 months post HCT. Routine monitoring of serum BK viral load is not recommended due to variable results. It can present with irritative and obstructive symptoms such as dysuria, painful micturition, pelvic pain, urinary obstruction and renal dysfunction. Management is with supportive care including antispasmodics such as oxybutynin or tolterodine and urinary analgesics such as phenzopyridine. High grade HC may need opioid analgesics for pain relief. Aggressive hydration with normal saline is needed to clear the hematuria and avoid blood clots and obstruction. High grade HC may need continuous bladder irrigation with cold normal saline using a three-way catheter. It is also essential to maintain platelets >50,000 and Hb > 8 in these patients. Antifibrinolytics are contraindicated as they can form clots and precipitate obstruction. In severe HC, intravenous cidofovir may have a role and has been used in various studies, however there is no standardized dosing schedule and prospective studies are needed to evaluate this (25). Adoptive cellular therapies using virus specific cytotoxic T-lymphocytes has been explored for severe and refractory cases of BK virus related HC, however it is not yet available on a commercial basis (26).

### Adenovirus

Human adenovirus (HAdV) infection is common in the first 6 months post allogeneic HCT, with an incidence of 14%–16% in the pediatric population (27). Most children have primary infection in infancy, presenting as flu-like illness or gastroenteritis, however the HAdV persists in T-lymphocytes and reactivation of infection occurs post T-cell depletion. It presents with fever, enteritis, elevated liver enzymes, and pancytopenia. Severe presentation can include hepatitis, myocarditis, encephalitis, pneumonia, multiorgan failure leading to death in disseminated cases. Hence, early diagnosis and initiation of treatment is critical in these patients. Risk factors include use of T-cell depletion methods pre-HCT (such as ex-vivo CD34+ selection or use of alemtuzumab or anti thymocyte globulin), grade III-IV GVHD, and donor source (haploidentical, cord blood, HLA mismatched donor) (27). The gastrointestinal tract acts as a reservoir for HAdV persistence and replication in children, hence stool adenovirus can be used for screening patients at risk for disseminated disease. Weekly blood and stool HAdV level monitoring is recommended for pediatric HCT recipients. HAdV viremia ≥10^3^ copies/ml or stool virus concentration above 10^6^ copies/g is indication for starting cidofovir at 5 mg/kg/week for 2 weeks followed by 3–5 mg/kg every 2 weeks. Treatment is continued till virological response (<400 copies/ml) and adequate immune reconstitution is achieved. This drug is associated with significant nephrotoxicity, and there have been significant development of adoptive cell therapy using third party virus-specific cytotoxic T-lymphocytes against HAdV. Randomized studies are ongoing for use of cell-based therapies against HAdV (26).

### Invasive fungal infections

Prolonged neutropenia, GVHD and immunosuppressive therapy are the predisposing factors for invasive fungal infections in HCT recipients, especially post-allogeneic transplant. Patients with prior history of invasive fungal infections warrant evaluation and secondary prophylaxis to prevent reactivation of the disease. Most frequently encountered invasive fungal infections are candidiasis and mold infection, Aspergillosis being the most common mold infection. Mucormycosis and Fusarium can also lead to life threatening invasive infections, requiring antifungals as well as surgical debridement in cases of sinus involvement. Fluconazole is started prophylactically pre-engraftment to prevent candidiasis. However, the 2009 ASBMT/EBMT guidelines recommend Posaconazole or voriconazole for antifungal prophylaxis in the setting of GVHD, and micafungin in the setting of prolonged neutropenia (28, 29).

## Gastrointestinal complications

### Sinusoidal obstructive syndrome (SOS)

Sinusoidal obstructive syndrome, also known as hepatic vaso-occlusive disease (VOD), is a life-threatening complication in recipients undergoing allogeneic and autologous HCT. SOS is estimated to occur in 20%–60% of children undergoing HCT, depending on age and underlying disease (30, 31). The European Society of Blood and Marrow Transplantation (EBMT) defines SOS as presence of two or more of the following: unexplained consumptive and transfusion-refractory thrombocytopenia, unexplained weight gain on three consecutive days despite use of diuretics or a weight gain >5% above baseline, hepatomegaly above baseline, ascites above baseline, and rising bilirubin from baseline on 3 consecutive days or bilirubin ≥2 mg/dl within 72 h (32).

The risk for SOS/VOD depends on type of transplant, with higher risk in allogeneic HCT, with unrelated and mismatched donors. Pre-transplant use of myeloablative conditioning including busulfan, and high dose total body radiation also increase the risk for VOD/SOS (33). In addition, underlying disease conditions such as primary hemophagocytic lympho-histiocytosis, adrenoleukodystrophy, osteopetrosis, thalassemia major, and high-risk neuroblastoma have an increased incidence of SOS/VOD. It is seen more commonly in younger children <2 years of age (31, 34) and in patients receiving gemtuzumab oogamicin and inotuzumab to treat acute leukemias. Previous hepatic disease and history of iron overload are also risk factors for SOS/VOD.

The highest risk of SOS/VOD is in the first month post-HCT. Hence, screening for SOS/VOD in the acute post-transplant period is vital, especially if any risk factors are present. This includes daily weights and close monitoring of fluid balance, overt edema, ascites, hepatomegaly, and jaundice. New onset transfusion-refractory thrombocytopenia not explained by other causes, such as sepsis, in the early period post-transplant, can be the earliest sign of SOS/VOD. Liver ultrasound often demonstrates decreased or reversed flow in the portal vein (35). Invasive methods such as liver biopsy and measuring hepatic venous gradient pressure are not recommended in the clinical setting due to the risk of bleeding (36). The use of ursodeoxycholic acid in preventing SOS/VOD is not well established. However, patients receiving this show decreased hepatotoxicity, decreased GVHD, and improved overall survival (37). A phase III randomized control trial showed the benefit of prophylactic defibrotide in reducing the incidence of SOS/VOD in patients <18 years of age (38, 39). Treatment of SOS/VOD involves symptomatic care, including oxygen, strict fluid, and electrolyte balance, peritoneocentesis, and hemodialysis as needed. Defibrotide is hypothesized to protect the hepatocytes and endothelial cells from injury and restores the coagulation balance. Multiple studies have shown improved complete response and overall survival when defibrotide is used as a treatment for VOD/SOS, and it is now FDA approved for use in this condition. Severe, untreated VOD/SOS has a mortality rate of >80%, resulting in multi-organ dysfunction, hence it is critical to identify and treat in transplant recipients.

### Neutropenic enterocolitis (typhlitis)

Neutropenic enterocolitis (NEC) is a severe inflammatory disorder of the intestines in patients with neutropenia. It is a critical condition with a high mortality if left undiagnosed. It is believed to occur due to translocation of gut bacteria through the friable intestinal mucosa damaged by the intensive chemotherapy. Patients usually present with abdominal pain, diarrhea, and fever. Fever may be absent in severe neutropenia. Severe cases can have hematochezia, abdominal distension, and paralytic ileus. Persistent NEC can lead to bowel perforation if unrecognized. CT scan classically shows bowel wall thickening, which is however nonspecific. Severe cases may show presence of pneumatosis intestinalis which is more suggestive of typhilitis. Management is supportive with bowel rest, intravenous fluids, parenteral nutrition, broad spectrum antibiotics, and correction of coagulopathy and thrombocytopenia to prevent bleeding. Routine use of granulocyte colony stimulating factor (G-CSF) is controversial in typhlitis. Early surgical consultation is indicated for all patients with NEC, with surgical intervention needed for bowel perforation, persistent gastrointestinal bleeding, or pneumoperitoneum.

## Neurological complications

Central nervous system (CNS) complications are common and life-threatening after allogeneic HCT. These can be infectious, metabolic, toxic, vascular, or immune mediated. Neuroimaging is crucial for early diagnosis and treatment, preferably MRI.

### CNS infections

Incidence of CNS infections is ∼4%, however mortality secondary to it can be as high as 67% (40). The most common CNS infections are opportunistic infections with toxoplasmosis and aspergillus. Early in transplant, due to decreased neutrophil counts, the vasogenic edema seen on MRI with CNS aspergillosis may be absent. Median onset of toxoplasmosis is 84 days post-transplant with MRI showing typical multifocal lesions with rim enhancement. Mortality can approach 71% if left undiagnosed. Bacterial meningitis and brain abscess is rare but life-threatening emergencies.

### Posterior reversible encephalopathy syndrome (PRES)

Posterior reversible encephalopathy syndrome is a rare but severe complication with vasogenic cerebral edema. PRES usually presents within the first month post-transplant with headaches, nausea, vomiting, seizures, visual disturbances, altered mental status, and focal neurological deficits. Acute hypertension may be noted. PRES is seen with allogeneic HCT, especially in myeloablative conditioning and with the use of calcineurin inhibitors such as tacrolimus and cyclosporine. It can also be seen secondary to infections and renal failure due to other transplant-related complications. Sickle cell disease has been reported as an independent risk factor for post-HCT PRES, however there was no association of prior history of cerebral vasculopathy or silent strokes with occurrence of PRES (41). The pathophysiology remains unclear but is potentially related to immune system activation, vascular instability, and endothelial dysfunction leading to cytokine release (42). MRI is the diagnostic modality of choice and most commonly shows symmetric cortical and subcortical hyperintense signals in bilateral parieto-occipital lobes (43, 44). Prompt diagnosis and treatment results in a complete reversal of this complication. If left unrecognized, patients can develop intracranial hemorrhage, ischemia, infarction, and even death. Treatment aims to treat the underlying cause, such as using alternate immunosuppressive agents, managing hypertension and seizures, and correcting electrolyte disturbances.

### Drug toxicity

High dose chemotherapy as well as supportive care treatment given during allogeneic and autologous HCT can result in many neurological complications including seizures, PRES, encephalopathy, neuropathy, progressive multifocal leukoencephalopathy, and idiopathic intracranial hypertension (Table 3). A high index of suspicion with prompt treatment is warranted for these complications.

**Table 3:** Potential neurological consequences with medications commonly used in HCT.

1. Seizures	Busulfan Cytarabine Melphalan Acyclovir Cefepime Imipenem Cyclosporine Tacrolimus Fludarabine
2. PRES	Carboplatin Etoposide Linezolid Cyclosporine Tacrolimus Sirolimus Fludarabine
3. Encephalopathy	Carmustine Etoposide Fludarabine Ifosfamide Melphalan Acyclovir Cefepime
4. Neuropathy	Carboplatin Etoposide Linezolid Posaconazole (especially with vincristine) Tacrolimus (optic neuropathy)
5. Progressive multifocal leukoencephalopathy	Alemtuzumab Fludarabine (cortical blindness) Mycophenolate
6. Idiopathic intracranial hypertension (pseudotumor cerebri)	Cyclosporine

### Intracranial hemorrhage

Subdural and subarachnoid hemorrhage are rare in the post-transplant setting. However, intraparenchymal hemorrhage is reported to have an incidence of 1%–2% and is associated with high mortality (45). Management is supportive with correction of any underlying coagulopathies and thrombocytopenia, and a team based approached with neurology and neurosurgery.

## Immunological complications

### Transplant-associated thrombotic microangiopathy (TA-TMA)

TA-TMA is an underrecognized complication post allogeneic and autologous HCT (46). TA-TMA is associated with the triad of endothelial cell activation, complement dysregulation, and microvascular hemolytic anemia. This can potentially lead to multiorgan failure and death if left untreated. In a large multicenter study, Dandoy et al. estimated an incidence of 16% in the pediatric and adolescent population, with the majority undergoing HCT for non-malignant diseases (46). Increased risk factors for TA-TMA include allogeneic HCT (47), use of calcineurin/mTOR inhibitors, presence of GVHD, venous thromboembolic disease, HLA mismatch, ABO incompatibility, and infections, especially viral and fungal (48). In addition, busulfan and cyclophosphamide-containing regimens have also been implicated in an increased incidence of TA-TMA. TA-TMA primarily affects the kidneys but can present with intestinal TMA, pulmonary hypertension, posterior reversible encephalopathy syndrome, and multiorgan dysfunction. Given the similar presentation to other complications associated with HCT, a high index of suspicion is needed for the timely diagnosis of TA-TMA. Earliest signs include hypertension, especially if refractory to two or more agents, and proteinuria as measured by urine protein-creatinine ratio (49). Intestinal TMA (iTMA) presents with severe abdominal pain, vomiting and diarrhea, GI bleeding, and ascites. iTMA is very difficult to differentiate from intestinal GVHD due to a lack of well-defined criteria differentiating the two entities and the co-existence of both conditions on diagnostic workup (50). However, diagnosis usually requires a biopsy and looking at the submucosal vasculature (51). CNS involvement is considered an unfavorable prognostic predictor and can present with headaches, seizures, altered sensorium, and delirium. Posterior reversible encephalopathy has been described in the pediatric population in the setting of TA-TMA-associated refractory hypertension (52). In the absence of GVHD, refractory and recurrent pleural and pericardial effusions should raise suspicion for TA-TMA (53). Pulmonary hypertension can present with unexplained hypoxemia. The potential use of echocardiography as a screening tool for early identification of vascular injury has been proposed. As seen in Table 4, a wide range of diagnoses can manifest similarly to TA-TMA post HCT, making it challenging to identify. Most cases are known to occur within 100 days of transplant. There are various proposed diagnostic criteria for TA-TMA. However, they are based on retrospective analyses and include non-specific markers such as serum haptoglobin levels and the presence of schistocytes in peripheral smear. There is a critical need for a unified diagnostic criterion to assist in the timely recognition of TA-TMA (54). The proposed criteria by Jodele et al. (49) has become the standard for diagnosis, using terminal complement levels, hypertension, proteinuria, and increased LDH as screening markers for early identification of TA-TMA. High-risk TMA is defined as nephrotic-range proteinuria (proteinuria ≥30 mg/dl × 2 or urine protein-creatinine ratio ≥2) and elevated sC5b-9 (>244 ng/ml) at the time of diagnosis, or the presence of one of these in the setting of multiorgan dysfunction (MODS).

**Table 4:** Differential diagnosis of TA-TMA.

Organ affected	Clinical features	Differential diagnosis	Diagnostic tests
Kidney	Hypertension (refractory), proteinuria, acute or chronic kidney injury	Medications (CNI, steroids, other nephrotoxic agents), infections (BK viremia, acute cystitis), engraftment syndrome	- Urinalysis - Urine protein/creatinine ratio - Urine BK virus
GI tract	Abdominal pain, diarrhea, vomiting, bleeding, ascites	GVHD, SOS, infections, medications, engraftment syndrome	- Liver function tests - Coagulation panel - Viral serologies - Stool culture - Amylase, lipase - Liver US - Endoscopy and biopsy to rule out GVHD
CNS	Headaches, seizures, hallucinations, confusion	Infection, delirium, fludarabine toxicity, engraftment syndrome	- Viral serologies - MRI brain - Thiamine and other nutritional deficiencies
Cardio-pulmonary	Pulmonary hypertension, refractory pleural and pericardial effusions	Infection, engraftment syndrome, GVHD, medication, radiation pneumonitis, idiopathic pneumonia syndrome, pulmonary VOD	- Chest Xray/CT - Pulmonary function test - Bronchoscopy - EKG, echocardiography

The approach to the treatment of TA-TMA is supportive care and targeted therapy. Supportive care includes preventative measures such as modifying choice of conditioning regimen, avoiding infections, minimizing transfusions, managing hypertension, and substituting CNI as GVHD prophylaxis with other agents. Targeted therapies include therapeutic plasma exchange, rituximab, defibrotide, and eculizumab. It has been seen that prompt identification and early initiation of treatment can lead to improved outcomes.

Eculizumab is a monoclonal anti-C5 (complement 5) antibody that blocks the terminal complement pathway and prevents the formation of membrane attack complex. It is considered the first-line treatment for TA-TMA, with dosing based on eculizumab trough and C50 levels. Although not FDA approved for TA-TMA, many retrospective and prospective studies have shown improved overall survival with eculizumab in this setting (55). The key is early initiation of treatment and achieving sustained therapeutic levels. Unlike atypical hemolytic uremic syndrome, these patients do not require life-long treatment with eculizumab since the trigger for endothelial dysfunction is temporary. However, treatment with complement-blocking agents increases the risk for Neisseria meningitides, so primary prophylaxis is recommended.

## Cytokine release syndrome (CRS)

Cellular immunotherapies based on T-cell engineering and chimeric antigen receptor (CAR) T-cell therapy for various oncological diseases have gained popularity in the past decade. CRS may occur in 54%–91% of patients receiving autologous CAR-T cells (56). However, current diagnostic criteria are based on clinical manifestations that may delay the diagnosis and treatment of CRS. In addition, haploidentical allogeneic peripheral blood HCT and virus specific T-cell therapies can also present with CRS and close monitoring is warranted.

Risk factors for CRS include pre-CAR T cell infusion treatments (salvage therapy between collection and infusion, intensity of lymphodepletion, use of fludarabine for lymphodepletion), leukemia burden before CAR-T cell therapy, infused dose of CAR-T cells, older patient age and severe thrombocytopenia (57).

CRS usually presents with fever, myalgia, arthralgia, nausea, vomiting, skin rash, hemodynamic instability, and capillary leak syndrome (hypotension, tachycardia, disseminated intravascular coagulation, and neurological toxicity) (57). Lee et al. proposed a grading system widely used for grading the severity of CRS (58) (Table 5).

**Table 5:** Grading system for CRS proposed by Lee et al.

Grading	Toxicity	Treatment
Grade 1	Fever, fatigue, headache, myalgia, malaise	• Supportive care • Antibiotics
Grade 2	• Oxygen requirement <40%. • Fluid responsive hypotension/sow dose single agent vasopressor • Grade 2 organ toxicity per CTCAE	• Supportive care • Antibiotics • Xygen • Fluids +/− low-dose vasopressors • +/− Tocilizumab (older patients/comorbidities)
Grade 3	• Oxygen requirement >40% • Hypotension needing high dose/multiple vasopressors • Grade 3 organ toxicity per CTCAE • Grade 4 transaminitis	• Supportive care • Antibiotics • Oxygen +/− pressor support • Tocilizumab • +/− high dose steroids
Grade 4	• Need for ventilatory support • Grade 4 organ toxicity (excluding transaminitis)	• Supportive care • Antibiotics • Steroids + Tocilizumab • +/− IL2R and TNF-α inhibitors

Tocilizumab is a humanized monoclonal antibody against soluble and membrane-bound IL-6 receptors and was FDA-approved in 2017 for treating CRS-related toxicities following CAR T-cell infusion (59). Tocilizumab does not cause significant loss of CAR T-cell activity and can produce a response within a few hours of the drug infusion. The preferred dose is 12 mg/kg for patients <30 kg, and 8 mg/kg for patients >30 kg. However, significant patients show resistance to tocilizumab (60).

Another monoclonal antibody against IL-6 is siltuximab, which has a higher affinity for IL-6 than tocilizumab. It is now used for patients resistant to steroids and tocilizumab. Other agents have been used with varying results in resistant CRS, such as TNF-α (etanercept and infliximab) and IL1R (anakinra) inhibitors (58). There are ongoing studies to use a “suicidal” gene construct to arm the T-cells that, when activated, can self-destruct the lymphocytes and limit the toxicity from immune hyperactivation. Recently developed split, universal and programmable (SUPRA) CAR system is a promising pre-clinical design that may help reduce the incidence of CRS without reducing the antitumor response of CAR T-cells (61).

## Graft-versus-host disease

GVHD is an immunological disorder that develops after transplantation when the donor or graft cells attack the recipient's tissues. Graft versus host disease (GVHD) continues to cause significant morbidity and mortality after allogeneic HCT. Acute GVHD (aGVHD) is seen in 30%–70% of recipients, while chronic GVHD occurs in 20%–50% of recipients based on the type of transplant, patient characteristics, and GVHD prophylaxis regimen (62). The most affected organs are the skin, gastrointestinal tract, liver, and lungs. The risk of GVHD in related transplants is ∼30%–40%, whereas it can be as high as 80% in unrelated transplants. Acute GVHD usually presents in the first three months post-transplant, while chronic GVHD can occur at any time after. For the purpose of this review, we will focus on acute GVHD as it can present as a life-threatening emergency.

The most frequent organs affected in aGVHD include the skin, gastrointestinal tract (GI tract), and liver. Clinical manifestations and stages of aGVHD are summarized in Table 6.

**Table 6:** Clinical manifestations and stages of aGVHD (65).

Organ	Stage	Clinical manifestations
Skin	0	No active(erythematous) GVHD rash
	1	Maculopapular rash <25% body surface area (BSA)
	2	Maculopapular rash 25%–50% BSA
	3	Maculopapular rash >50% BSA
	4	Generalized erythroderma (>50% BSA) plus Bullous formation and desquamation >5% BSA
Upper GI	0	No or intermittent nausea, vomiting or anorexia
	1	Persistent nausea, vomiting or anorexia
Lower GI (stool output/day)	0	<10 ml/kg/day or <4 episodes
	1	10–19.9 ml/kg/day or 4–6 episodes/day
	2	20–30 ml/kg/day or 7–10 episodes/day
	3	>30 ml/kg/day or >10 episodes/day
	4	Severe abdominal pain with or without ileus, or grossly bloody stool (regardless of stool volume)
Liver	0	Bilirubin <2 mg/dl
	1	Bilirubin 2–3 mg/dl
	2	Bilirubin 3.1–6 mg/dl
	3	Bilirubin 6.1–15 mg/dl
	4	Bilirubin >15 mg/dl

Skin involvement is usually the earliest and most recurrent manifestation of acute GVHD. The inflammation can cause pruritis or severe sunburn. A biopsy is typically necessary for confirmation of diagnosis. Involvement of the GI tract can present with diarrhea, nausea, vomiting, dyspepsia, anorexia, and food intolerance. There can be possible mucositis and gingivitis. Biopsies taken from the lower and upper GI tract are needed to confirm the diagnosis (63). Involvement of the liver can present with nausea, fever, anorexia, painful hepatomegaly, pale stool, dark urine, abnormal liver functions, and increased cholesterol levels.

Corticosteroids remain the first line treatment for aGVHD despite suboptimal responses of 40%–60% (64). Steroid-refractory (SR) aGVHD has a poor prognosis with a mortality rate of 70%–80%. This is because of low response rates with second-line treatments. No second-line therapy has been shown to be superior to another in SR aGVHD, and treatment choice is based on the patient, anticipated side effects, and physician preference. Other treatments that have been studied include JAK1/2 inhibitor (Ruxolitinib), proteosome inhibitor (Bortezomib), and monoclonal antibodies (Vedolizumab, Natalizumab).

## Pulmonary complications

### Idiopathic pneumonia syndrome

Idiopathic pneumonia syndrome (IPS) is a noninfectious pulmonary complication seen post HCT due to diffuse lung injury. Although the incidence is reported between 4%–12%, the mortality associated with this rare entity can be as high as 60%–86% despite aggressive treatment (65). It is most seen in the first 30 days post HCT. Risk factors include underlying hematological malignancy, high-dose TBI, grade III-IV acute GVHD and older age. Lung injury due to myeloablative conditioning, immunological cell-mediated and cytokine injury, and occult pulmonary infections are the likely causative events for IPS. Need for mechanical ventilation and renal insufficiency have poor prognostic outcomes in patients with IPS. Treatment is primarily supportive and use of high dose steroids (2–4 mg/kg/day), however there is no clear evidence of benefit of steroids in IPS.

### Diffuse alveolar hemorrhage

Diffuse alveolar hemorrhage (DAH) is a serious pulmonary complication after HCT, with an incidence of 3%–10% and a mortality of 70%–100%, most commonly due to respiratory failure, multiorgan failure and sepsis (66). The exact pathogenesis is unknown, but it is hypothesized to result from damage to the pulmonary microcirculation secondary to inflammation and cytokine release post HCT. DAH is similar to IPS, however there is minimal fibrosis despite the inflammation in DAH, whereas IPS is associated with dysregulated wound healing and pulmonary fibrosis. DAH occurs mostly in the first 30 days post HCT, and is presents with fever, hypoxemia, cough and hemoptysis. Chest radiograph shows bilateral ground glass opacities and patchy consolidation. Bronchoalveolar lavage is diagnostic with presence of bloody fluid and >20% hemosiderin laden alveolar macrophages. Risk factors for DAH include TBI, severe GVHD, use of sirolimus or defibrotide, use of umbilical cord graft and graft failure. TMA associated renal insufficiency can also lead to DAH. Most patients with DAH need intensive care and invasive mechanical ventilation with high positive end expiratory pressures (PEEP) or high frequency oscillatory ventilation (HFOV). Supportive measures such as prophylactic antibiotics, fluid and electrolyte management and correction of coagulopathies are the mainstay of treatment. High dose steroids are used for short duration given the underlying inflammatory pathophysiology. Antifibrinolytic agents such as aminocaproic acid and nebulized tranexamic acid have been reported in small cohorts with some success in cessation of bleeding. Use of intrapulmonary recombinant factor VII has been reported with encouraging results, however prospective studies are needed to evaluate its use and standardize dosing. Transfusions may increase the risk for transfusion-related acute lung injury (TRALI) and thromboelastography (TEG) and rotational thromboelastometry (ROTEM) should be used to guide transfusion of platelets/blood products.

## Conclusion

We have attempted to present a narrative review and not a systemic review or meta-analysis, which are the limitations of this manuscript. However, with the increasing use of HCT, there is a rising need for early identification and treatment of complications seen with HCT. There have been dramatic advances in managing these complications but we still have a long way to go. In addition, there is a demand for developing treatments that can be used to prevent these complications and tissue damage without being immunosuppressive. Many novel agents are under study and will hopefully provide improved symptoms, prevent the progression of these complications, enhance patient quality of life, and ultimately improve overall survival.
